# Sex-Related Differences in White Matter Asymmetry and Its Implications for Verbal Working Memory in Psychosis High-Risk State

**DOI:** 10.3389/fpsyt.2021.686967

**Published:** 2021-06-14

**Authors:** Saskia Steinmann, Amanda E. Lyall, Mina Langhein, Felix L. Nägele, Jonas Rauh, Suheyla Cetin-Karayumak, Fan Zhang, Marius Mussmann, Tashrif Billah, Nikos Makris, Ofer Pasternak, Lauren J. O'Donnell, Yogesh Rathi, Marek Kubicki, Gregor Leicht, Martha E. Shenton, Christoph Mulert

**Affiliations:** ^1^Psychiatry Neuroimaging Branch, Department of Psychiatry and Psychotherapy, University Medical Center Hamburg-Eppendorf, Hamburg, Germany; ^2^Psychiatry Neuroimaging Laboratory, Department of Psychiatry, Brigham and Women's Hospital, Boston, MA, United States; ^3^Department of Psychiatry, Massachusetts General Hospital Harvard Medical School, Boston, MA, United States; ^4^Department of Radiology, Brigham and Women's Hospital Harvard Medical School, Boston, MA, United States; ^5^Center for Psychiatry and Psychotherapy, Justus-Liebig-University Giessen, Giessen, Germany

**Keywords:** sex differences, white matter asymmetry, high risk of psychosis, working memory, diffusion-tensor-imaging

## Abstract

**Objective:** Sexual dimorphism has been investigated in schizophrenia, although sex-specific differences among individuals who are at clinical high-risk (CHR) for developing psychosis have been inconclusive. This study aims to characterize sexual dimorphism of language areas in the brain by investigating the asymmetry of four white matter tracts relevant to verbal working memory in CHR patients compared to healthy controls (HC). HC typically show a leftward asymmetry of these tracts. Moreover, structural abnormalities in asymmetry and verbal working memory dysfunctions have been associated with neurodevelopmental abnormalities and are considered core features of schizophrenia.

**Methods:** Twenty-nine subjects with CHR (17 female/12 male) for developing psychosis and twenty-one HC (11 female/10 male) matched for age, sex, and education were included in the study. Two-tensor unscented Kalman filter tractography, followed by an automated, atlas-guided fiber clustering approach, were used to identify four fiber tracts related to verbal working memory: the superior longitudinal fasciculi (SLF) I, II and III, and the superior occipitofrontal fasciculus (SOFF). Using fractional anisotropy (FA) of tissue as the primary measure, we calculated the laterality index for each tract.

**Results:** There was a significantly greater right>left asymmetry of the SLF-III in CHR females compared to HC females, but no hemispheric difference between CHR vs. HC males. Moreover, the laterality index of SLF-III for CHR females correlated negatively with Backward Digit Span performance, suggesting a greater rightward asymmetry was associated with poorer working memory functioning.

**Conclusion:** This study suggests increased rightward asymmetry of the SLF-III in CHR females. This finding of sexual dimorphism in white matter asymmetry in a language-related area of the brain in CHR highlights the need for a deeper understanding of the role of sex in the high-risk state. Future work investigating early sex-specific pathophysiological mechanisms, may lead to the development of novel personalized treatment strategies aimed at preventing transition to a more chronic and difficult-to-treat disorder.

## Introduction

Sexual dimorphisms in schizophrenia have been well-documented in a range of areas including symptomatology, treatment response, and outcome. However, one of the most robust findings is the earlier onset of illness in males (1). More specifically, age of onset in males tends to occur in late teens to early 20s, whereas females typically show an onset to illness ~3–5 years later (20–30 years of age), with another period of conversion to illness observed just after the onset of menopause (40–50 years of age) (2, 3). Females have also been shown to respond better to treatment, to recover to a greater degree even in the absence of medication (4), and to show a more benign course of illness (1). Since much of this research has been conducted with individuals with established diagnoses, there is less known about sex-specific differences in those individuals who are at clinical high-risk for developing psychosis (CHR). Given the demonstrated impact of sex on illness course and outcome, the investigation of sex-specific pathologies in CHR individuals could lead to the identification of biomarkers which might be crucial for developing more effective sex-specific therapeutic interventions.

A promising indicator of a CHR state for psychosis is working memory impairments (5–7). Specifically, deficits in verbal working memory have been repeatedly shown to be more severe in CHR individuals who later convert to psychosis compared to those who do not (8, 9). Exploring verbal working memory with imaging (10), and lesion studies (11), reveal a core frontoparietal network of slightly left-lateralized activation, which involves primarily dorsolateral prefrontal and posterior parietal cortices (12). These brain regions are connected via two key white matter association fibers, the superior longitudinal fasciculus (SLF) and the superior occipitofrontal fasciculus (SOFF) (13, 14). White matter maturation and white matter microstructure of the SLF, in particular, have been associated with verbal working memory performance in healthy children, adolescents, and adults (15, 16), as well as in patients with recent-onset schizophrenia (17).

Verbal memory impairments in patients with schizophrenia have also been linked to white matter tract asymmetries. Tract asymmetries in this study are defined as the relative ratio of white matter microstructural measurements, such as fractional anisotropy (FA), a measure of the directionality of white matter fiber bundles, in white matter tracts in the left hemisphere compared to the right hemisphere (18). Since the left and right hemispheres are not identical, such asymmetries can provide important functional information. For example, a left > right asymmetry in several tracts, particularly those of the SLF, has been consistently reported in healthy adults (19, 20) and in the developing human brain (21, 22). However, early stage/course patients with schizophrenia tend to show a stronger rightward asymmetry in the SLF (23). Comparable reductions in left > right asymmetry have also been reported in populations of chronic patients for the SLF (24) and for other components of the frontoparietal network underlying verbal working memory, specifically the SOFF (25), and the inferior fronto-occipital fasciculus (IOFF) (26). Very recently, it has been demonstrated that even the emergence of specific symptoms such as auditory-verbal hallucinations in schizophrenia is related to longer left than right-sided arcuate fasciculus fiber tracts (27). Taken together, there is reliable evidence that individuals with schizophrenia tend to show deviations in hemispheric laterality (i.e., cerebral asymmetry, left hemispheric dominance for language, and handedness) from healthy individuals (28). For instance, several studies reported less left-lateralized language functions (29) as well as a higher rate of non-right-handedness (i.e., mixed- and left-handedness) in schizophrenia patients (30, 31) and their healthy relatives (32, 33). Hence, a genetic link has been suggested between atypical laterality and schizophrenia (34).

While white matter tract asymmetries have also been reported to increase with a longer duration of illness (35), it has been suggested that their origins are the result of neurodevelopmental abnormalities in schizophrenia (24, 36). Accordingly, further investigations of these possible neurodevelopmental asymmetries in those at CHR for developing schizophrenia is needed.

Sex has also been shown to be a major influence on the development of white matter tracts (37, 38). For example, males show a later onset in the maturation of white matter tracts than do females (39, 40). Moreover, this finding has been linked to an earlier onset of pubertal maturation in females (~2–3 years earlier) (41). However, the role of sex on white matter tract asymmetries in verbal working memory in CHR individuals is not as yet known. Demonstrating such sex-specific neurodevelopmental disturbances in the working memory-relevant white matter network of CHR individuals may be central to understanding the emergence of psychosis symptomatology.

Given that verbal working memory deficits have been shown in patients with schizophrenia and are a promising symptom criterion for CHR conversion to psychosis, we investigated possible alterations in sexually dimorphic asymmetry in four white matter tracts in CHR for psychosis individuals compared to healthy controls (HC). The tracts of interest were central components of the verbal working memory circuit, including the SLF with its three subcomponents (SLF-I, II and III), in addition to the SOFF. In this study, we employed a novel data-driven fiber clustering approach based on diffusion magnetic resonance imaging (dMRI), and an anatomically curated white matter fiber clustering atlas (42). We hypothesized that: (1) CHR females show a greater rightward asymmetry of SLF, and (2) that these alterations would correlate with verbal working memory dysfunctions.

## Materials and Methods

### Ethics Statement

The present study was part of a larger ongoing project within the context of the Collaborative Research Center 936 (“multi-site communication in the brain,” www.sfb936.net) investigating functional and structural connectivity in schizophrenia. Parts of this data were already published (43). The study was approved by the Ethics Committee of the Medical Association Hamburg and was performed in accordance with the seventh version of the Declaration of Helsinki ethical standards. All participants provided written informed consent (or in case of minors from their respective legal guardian).

### Participants

Our participant sample consisted of 29 right-handed individuals at CHR for psychosis (12 men, mean age 21.00 ± 4.14, 17 women, mean age 20.71 ± 3.29) and 25 HC. The participants' handedness was verified with the empirically validated Edinburgh Handedness Inventory (44). Four HCs were excluded, two as a result of severe signal dropouts, one as a result of presence of a hydrocephalus, and one as a result of sex transformation, resulting in 21 HC (10 men, mean age 23.00 ± 4.85, 11 women, mean age 22.55 ± 3.53). The exclusion criteria for all participants were defined as follows: (1) presence of major neurological disorder or head injury, (2) current substance abuse or dependence, (3) intellectual disability (IQ <70), and (4) left-handedness. Additionally, HCs were excluded if they had a (5) history of psychotic disorders or if they had been (6) previously diagnosed with any psychiatric disorder. The screening for eligibility was carried out in the form of a semi-structured interview by a clinical psychiatrist or psychiatry resident with a minimum of 4 years of clinical experience.

The inclusion criteria for CHR for psychosis were defined in line with the criteria of the Early Detection and Intervention Program of the German Research Network on Schizophrenia (GNRS) (45), including the following: presence of either (1) basic symptoms syndrome (BSS), (2) a schizotypal personality disorder/ plus a decline in global functioning (GRD), (3) attenuated psychotic symptoms (APS), or a brief limited intermittent psychotic syndrome (BLIPS). Sociodemographic and clinical data for 29 CHR individuals and 21 HC are presented for females and males separately in Table 1.

**Table 1:** Sociodemographic and clinical characteristics of the sex-related subgroups.

	**HC**		**CHR**		**HC vs. CHR**
	**Females (*n* = 11)**	**Males (*n* = 10)**	**Females (*n* = 17)**	**Males (*n* = 12)**	***p*-values**
	**M ± SD (*N*)**	**M ± SD (*N*)**	**M ± SD (*N*)**	**M ± SD (*N*)**	
Age (years)	22.55 ± 3.53	23.00 ± 4.85	20.71 ± 3.29	21.00 ± 4.14	0.178
Educational years	15.00 ± 2.36	14.05 ± 3.21	13.25 ± 2.96	12.66 ± 2.89	0.094
CHR syndrome:	n.a.	n.a.			n.a.
APS			6	5	
APS + BLIPS			1	1	
APS + BS			2	3	
APS + GRD			1	0	
BLIPS			1	0	
BLIPS + GRD			1	0	
GRD			0	1	
BS			4	1	
BS + GRD			0	1	
APS + BS + BLIPS + GRD			1	0	
Laterality index of SLF-III	0.0002 ± 0.036	0.022 ± 0.027	0.030 ± 0.028	0.042 ± 0.038	0.012^a^
Number of streamlines right SLF-III	709 ± 359	939 ± 683	1,049 ± 433	1,333 ± 559	0.05
Number of streamlines left SLF-III	743 ± 467	914 ± 643	931 ± 564	870 ± 750	0.467
Digit span backward	6.7 ± 1.25 (10)	7.20 ± 1.62	6.73 ± 2.18 (15)	6.50 ± 1.50 (11)	0.609 0.826^a^
Letter-Number Sequencing	15.77 ± 2.33 (9)	16.1 ± 2.96	16.00 ± 2.96 (14)	13.66 ± 3.36	0.07 0.004^a^
PANSS scores:	n.a.	n.a.	(15)		
Positive			13.2 ± 3.42	11.58 ± 4.92	0.347^b^
Negative			8.92 ± 2.31	11.5 ± 3.77	0.236^b^
Disorganization			13.2 ± 4.27	13.41 ± 2.77	0.881^b^
Excitement			10.06 ± 1.86	10.91 ± 3.57	0.433^b^
Distress			17.53 ± 5.54	14.5 ± 4.12	0.127^b^
SOPS:	n.a.	n.a.			
Positive			6.78 ± 5.50	5.41 ± 4.73	0.507^b^
Negative			4.76 ± 5.05	5.16 ± 4.58	0.934^b^
Disorganization			2.71 ± 2.86	2.08 ± 1.88	0.400^b^

a *Comparison between HC and CHR females*.

b *Comparison between CHR males and CHR females*.

*APS, Attenuated Positive Symptoms; BIPS, Brief Intermittent Psychotic Syndrome; GRD, Genetic Risk and Deterioration; BS, Basis Symptoms; PANSS, Positive and Negative Symptom Scale; SOPS, Scale of Prodromal Symptoms; HC, Healthy Controls, CHR, Individuals at Clinical High Risk for Psychosis; SD, Standard Deviation; N, Number of subjects; N.S., Not significant*.

### Cognitive and Clinical Assessments

All CHR individuals underwent the Mini Neuropsychiatric Interview (46) to record current psychiatric comorbidities and to ensure that CHR subjects had no schizophrenia spectrum disorder in the past. Psychopathology in CHR was assessed with the Positive and Negative Syndrome Scale (PANSS) (47) and the Scale of Prodromal Symptoms (SOPS) (48). PANSS scores were derived for positive, negative, disorganization, excitement and distress factors from the scale (49). Participants also underwent neuropsychological testing with an extensive battery including tests of memory, attention and executive functioning. Given this study's purpose, we focused on the measures relevant to verbal working memory, such as the Digit Span Backwards Task and the Letter-Number Sequencing Test of the Wechsler Adult Intelligence Scale (50). Neurocognitive performance data were available from 20 HC and 27 CHR (see Table 1).

### Diffusion Weighted Images

All images were obtained using a Siemens 3T scanner (Magnetom Trio) at the University Medical Center Hamburg-Eppendorf with a 12-channel head coil. Diffusion-weighted images (DWI) were acquired using an echo-planar imaging sequence (EPI) with the following parameters: TR = 7.7 s, TE = 85 ms, flip angle = 90°, 32 interleaved slices, slice thickness = 2 mm, field of view (FOV) = 216 × 256 mm^2^ matrix = 108 × 128, 30 non-colinear gradient directions with b = 1,000 s/mm^2^ collected twice and 10 images with b = 0 s/mm^2^. The quality control steps comprised visual inspection of the images for movement artifacts, EPI distortions and structural abnormalities using 3D Slicer (software version 4.5; www.slicer.org) (51). Gradient directions that showed severe artifacts or signal dropouts were removed after a careful quality control process. All scans were corrected for motion and eddy currents by affine registration of all volumes to the first B0 of the acquisition using the Linear Image Registration Tool (FLIRT), provided in the Oxford Center for Functional MRI of the Brain Software Library (FSL, http://fsl.fmrib.ox.ac.uk/fsl/). The gradient vector table was updated accordingly. A relative motion parameter was obtained for each scan from the transforms by calculating the average displacement between adjacent volumes. Finally, brain masks for skull stripping were generated automatically within 3D Slicer using Otsu's method and then manually edited to ensure anatomical accuracy. After preprocessing, each gradient was averaged from both acquisitions using an in-house developed script (52).

### Unscented Kalman Filter Tractography

Next, whole brain tractography was computed using the unscented Kalman filter tractography method (UKFt), as implemented in the open-source software package (https://github.com/pnlbwh/ukftractography). The UKFt fits a mixture model of compartments to the dMRI data while tracking fibers, taking advantage of information from the previous step along the fiber to help stabilize model fitting (53, 54). UKFt has been shown to be highly consistent in fiber tracking across age, state of health, and image acquisition (42), and superior than standard single-tensor tractography, in particular in the presence of crossing fibers and edema (55). In this study, we used a three-compartment model including two tensor compartments to model the fiber being tracked, and a potential additional fiber crossing it, and a third compartment of an isotropic tensor with diffusivity equal to that of extracellular water. The third compartment is aimed at modeling partial volume with cerebrospinal fluid (CSF) and other freely diffusing extracellular water pools (56, 57). The fibers were quantified using FA of tissue of the principal compartment (the one driving the direction of the UKFt), which is inherently corrected for crossing fibers or for partial volume with free-water. Tractography was seeded with 10 seeds per voxel in all voxels within the binary brain mask where a DTI fit yielded FA >0.08. Tracking stopped where the FA value of the principal compartment fell below 0.08, or the generalized anisotropy fell below 0.08.

### Automatic Identification of SOFF, SLF-I, SLF-II, and SLF-III

**Figure 1:**
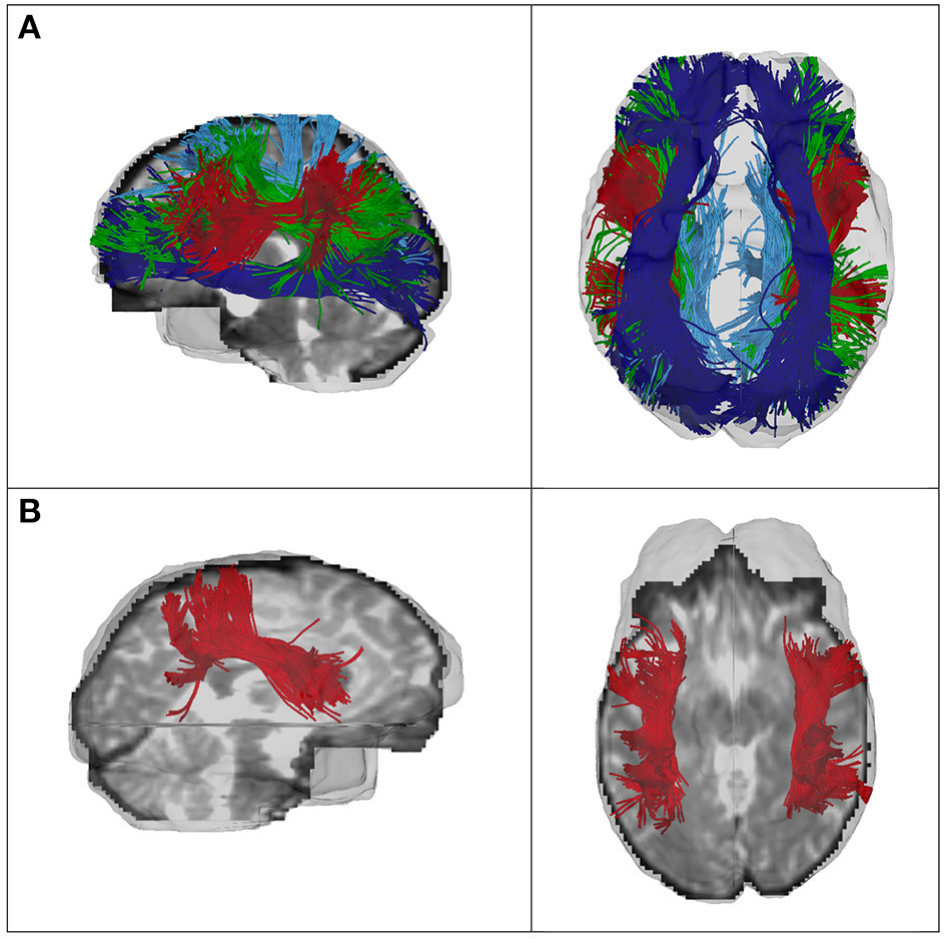
**(A)** Fiber clustering results of the SLF-I (light blue), SLF-II (green), SLF-III (red), and SOFF (dark blue) of one CHR female, and **(B)** of the SLF-III of one HC female. Left column, sagittal view; right column, axial view.

After performing whole-brain tractography using the UKFt + FW method, automated fiber parcellation was performed using tract registration and clustering methods (58, 59). Subject-specific bilateral SOFF, SLF-I, SLF-II, and SLF-III clusters were automatically identified according to the anatomically curated fiber clustering atlas, provided by the O'Donnell Research Group (ORG) (42) (http://dmri.slicer.org/atlases/). Recent studies have shown that this fiber clustering approach enables reliable fiber quantification and three-dimensional visualization (42, 60–62). The ORG atlas is based on 100 subjects, containing 800 cluster parcellations of the entire brain white matter and an anatomical tract parcellation that defines 58 deep white matter tracts, as well as 198 short and medium range superficial fiber clusters (42, 59, 63). For clustering, each fiber is compared to a variety of fibers in the atlas, giving a feature vector or “fingerprint” that is used to classify the fiber into a cluster. The fiber clustering result of one SLF-III tract is shown in Figure 1. To provide sufficient data quality, we excluded data from subsequent analysis for any tract with fewer than five streamlines. Finally, mean FA values were extracted for each fiber tract and exported to SPSS. All software used is publicly available, including computational tractography, fiber cluster analysis (https://github.com/SlicerDMRI/whitematteranalysis), as well as tractography visualization with anatomical hierarchies in 3D Slicer via the SlicerDMRI project (dmri.slicer.org) (64, 65).

### Laterality Index of SOFF, SLF-I, SLF-II, and SLF-III

Next, hemispheric asymmetry was calculated for all four tracts of interest using the laterality index (LI) based on the following formula: LI = (Right FA – Left FA)/(Right FA + Left FA) of the average FA across each tract. This index yields positive values for right-lateralized, negative values for left-lateralized white matter tracts, and zero values for absolute symmetry.

### Statistical Analysis

Group differences were compared by means of independent *t*-tests or Mann-Whitney-*U*-tests (depending on normal distribution and corrected for multiple comparison for demographic, clinical and behavioral data).

A linear mixed-effects (LMEs) model was used to analyse the effects of group, sex, white matter tracts and any group-related interactions on LI. LMEs have several advantages over traditional repeated-measures designs, as they can accommodate departures from assumptions of homogeneity of regression slopes and independence, and thus are better suited to model inter-individual variability (66). Moreover, LMEs are able to handle an unequal number of within-subject data (67), which is particularly important in the current study as we excluded one subject's white matter tract (i.e., SOFF) due to an insufficient number of streamlines. In this study, the model included Group (2 levels: CHR and HC), White Matter Tracts (4 levels: SOFF, SLF-I, SLF-II and SLF-III), and Sex (2 levels: female and male) as the fixed-effects factors, Subject as a random-effects factor (to account for within-subject variability), and FA-based LI as the dependent variable. To capture effects of possible confounding variables, our model also included age, years of education and their respective interaction with Group as fixed-effects. All the analyses were performed with SPSS version 25 (IBM SPSS Statistics for Macintosh).

## Results

### Sociodemographic and Clinical Variables

Sociodemographic and clinical variables of the sample are shown in Table 1. No differences were observed between CHR and HC in age, sex and years of education (all *p* > 0.09), nor between the sex-specific subgroups (all *p* > 0.15). Additionally, there were no significant differences in PANSS sub-scores, nor in SOPS sub-scores, between CHR males and CHR females (all *p* > 0.13). There were also no significant differences between group (CHR or HC) and gender (male or female) in working memory performances (all *p* > 0.15).

### Sex-Related Differences in Asymmetry of White Matter Tracts

**Figure 2:**
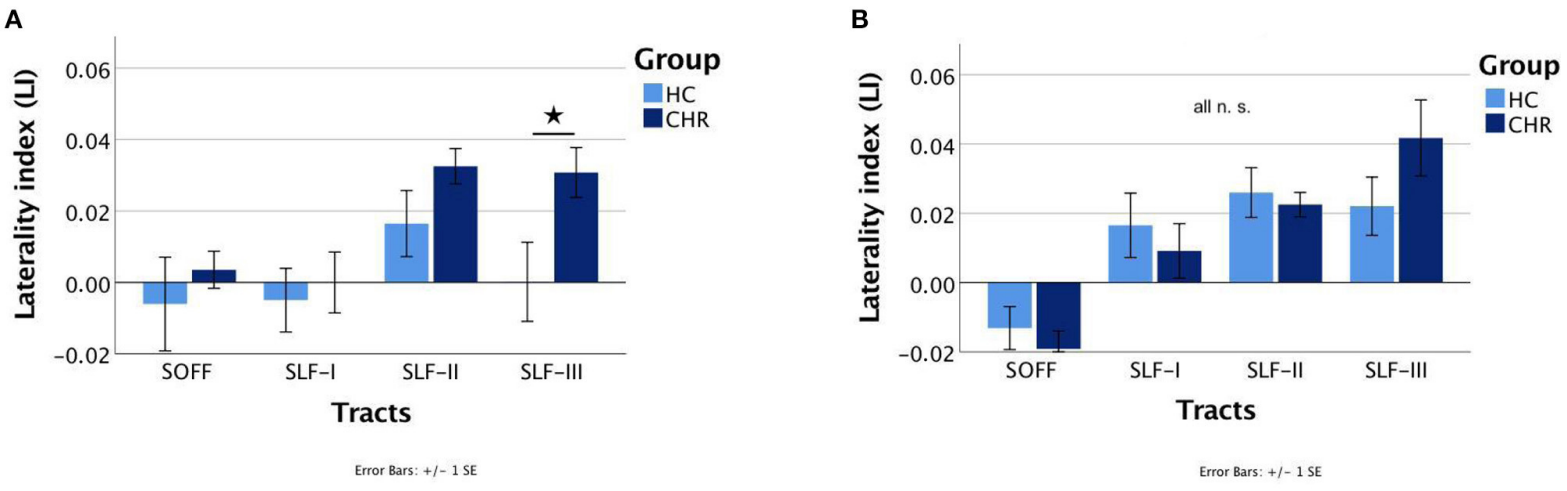
Mean laterality indices of the superior occipitofrontal fasciculi (SOFF) and the superior longitudinal fasciculi (SLF) I, II, and III for healthy controls (HC) and individuals at clinical high-risk (CHR). The significant different laterality index of the SLF-III between HC and CHR females is marked with an asterisk. **(A)** Females and **(B)** Males. N.S., not significant.

The interaction of Group × Sex had a significant effect on LI [F_(1, 48.574)_ = 4.099, *p* = 0.048], indicating that there are sex-related differences in terms of hemispheric asymmetry across the two groups (i.e., HC vs. CHR). Specifically, subsequent *post-hoc* pairwise comparisons within each group (HC or CHR) revealed a greater hemispheric asymmetry in healthy males than healthy females, but that did not reach the significance threshold (*p* = 0.051, Cohen's *d* = 0.35). There was no such difference between CHR males and CHR females (*p* = 0.45). Moreover, *post-hoc* pairwise comparisons within each sex demonstrated a significantly different LI between HC and CHR females (*p* = 0.008, Cohen's *d* = 0.49), but not between HC and CHR males (*p* = 0.91). To determine which tract was driving the effect in females, *post-hoc* pairwise comparisons between each white matter tract's LI revealed a significantly greater rightward asymmetry of the SLF-III in CHR females compared to HC females (*p* = 0.012, Cohen's *d* = 0.92) (Figure 2). There was no significant difference in any of the other three white matter tracts (all *p* > 0.1).

### Associations Between SLF-III Asymmetry and Verbal Working Memory

**Figure 3:**
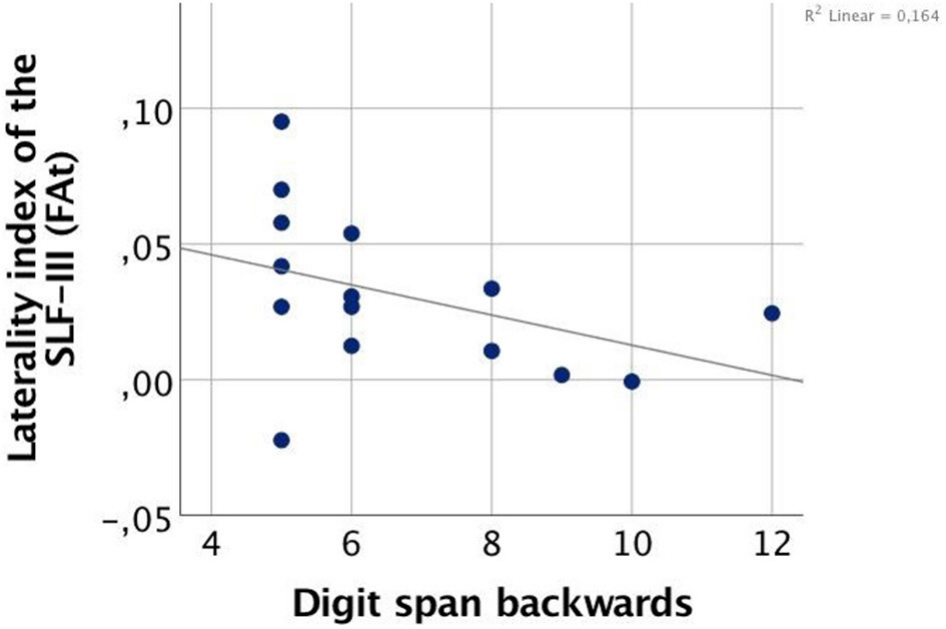
Correlation between FA-based laterality index of the superior longitudinal fasciculus (SLF) III and working memory performance in clinical high-risk females (CHR).

Correlation analyses were performed for SLF-III, which was the only tract with a significant group effect in hemispheric laterality. In CHR females, the laterality index of SLF-III correlated negatively with Digit Span Backwards performance (two-tailed Spearman's rho = −0.520, *p* = 0.047), indicating that in females, a greater degree of rightward asymmetry was related to poorer performance (Figure 3). No significant correlations were found in HC males, HC females, or CHR males.

## Discussion

In this study, we employed a state-of-the-art fiber clustering method to investigate the impact of sex on hemispheric white matter asymmetries within a verbal working memory circuitry in a population of individuals at CHR and matched HC. We observed a greater right > left asymmetry of the SLF-III in CHR females only. Further, we found that the greater degree of rightward asymmetry in the CHR females was related to poorer performance in the Digit Span Backwards Task of the WAIS. This relationship was not observed in the male CHR group nor in the male or female HC groups. Thus, this study reports an association between a putative sex-specific neurodevelopmental disturbance in the frontoparietal network and verbal working memory in female CHR individuals.

Our primary finding of aberrant white matter asymmetry in the SLF-III in female CHR individuals aligns well with earlier studies reporting decreased left > right asymmetry in first-episode and chronic patients with schizophrenia (23–26, 35). In this study, we chose to focus on white matter tract asymmetry in a frontoparietal network proposed to underlie verbal working memory because both have been previously considered core features of schizophrenia pathology (68, 69). For example, a reduction of the typical left > right asymmetry has been shown for frontoparietal tracts, such as the SLF (23, 24), the SOFF (25), and the IOFF (26) in patients with chronic schizophrenia.

Moreover, abnormalities within the SLF have been the most frequently reported alterations in CHR individuals (17, 70, 71), suggesting that alterations in the SLF precede illness onset. As stated previously, changes in white matter asymmetry have been associated with aberrant white matter development (24). Of further note, the SLF is one of the most dynamically changing white matter tracts from childhood to early adulthood (72). There is also evidence that the FA values of the SLF increase substantially between 8 and 22 years of age (73) and peak on average between 15 and 20 years of age (74) in healthy individuals. As this period overlaps with the onset of puberty, many have investigated the possible role that sex, as well as pubertal hormones, may have on the maturation of white matter (38, 75). Indeed, it has been consistently reported that white matter growth during puberty and adolescence follows a striking sexual dimorphism: age-related increases in white matter occur much more steeply in boys when compared to girls (76, 77). This faster degree of growth has been shown to be driven—at least in part—by testosterone in boys, but not in girls (38). Girls, on the other hand, show a negative relationship between estradiol and FA (37). In our sample, the average age of our CHR females is 21 years of age, placing them past the previously reported maturational peak for the SLF. When taken together, these findings suggest that our finding of a greater right > left asymmetry of the SLF-III in young adult CHR females only, might reflect a sex-specific deviation in white matter maturation and early development.

However, even in the presence of significantly altered white matter asymmetry, the CHR females in our sample still performed at a similar level to the female and male controls in the Digit Span Backwards task. This finding is also particularly interesting in light of the inverse relationship we observed between task performance and the degree of asymmetry in the CHR females. The Digit Span Backwards Task measures verbal working memory, by requiring temporary storage and manipulation of verbal information no longer available in the external environment (78). In the literature, worse performance within the Digit Span Backwards Task has been reported in first-degree relatives (79) and in patients with schizophrenia (80) compared to healthy controls. However, these studies tend not to control for sex, or they have limited power to test for sex differences. When taking sex into account, female patients perform better than males in the digit symbol subtest (81) and show, in general, higher levels of functioning in language, and in executive and memory domains (1).

Our study shows a similar behavioral trend, yet interestingly, we observed that female CHR individuals also exhibit greater structural differences than their male CHR counterparts. Though speculative, our finding of a greater right > left asymmetry of the SLF-III in CHR females may reflect a compensatory mechanism. Other studies have also described potential compensatory mechanisms in the brain of individuals with psychosis or of healthy controls at higher age (i.e., range: 64–78), while few have investigated the role that sex may play. For example, Di Biase and colleagues suggest that increases in FA in early stage psychosis patients may compensate for cortical thinning that occurs with longer durations of illness (82). Similarly, neural and cognitive decline in elderly subjects are thought to be compensated for by recruitment of homologous brain areas in the contralateral hemisphere (83). For example, the typical left-lateralized verbal memory network in young adults begin to appear more bilateral with increased age (84). Accordingly, we propose that the greater right > left asymmetry of the SLF-III of CHR females may be the result of developmental compensation for compromised neural processing in the left hemisphere in the female CHR individuals.

Moreover, we found no significant difference in white matter asymmetry in CHR males compared to HC males, which aligns well with another DTI study which also reports no significant white matter asymmetry differences in ultra-high-risk males who later developed psychosis (85). Thus, overall, hemispheric volume is a sexually dimorphic trait, i.e., the male brain tends to be more asymmetric than the female brains, a pattern replicated in healthy controls in the present study.

Concerning methodological aspects of the study, the present study demonstrates the efficacy and sensitivity of using and automatically annotating fiber clustering method for white matter tract parcellation across different populations. This method has been increasingly and successfully applied to clinical studies (42, 60, 61) and is shown to be superior to cortical-parcellation-based methods (62).

Several potential limitations of our study should be mentioned. Due to difficulty in recruiting CHR individuals, the sample size is not large, especially considering the sex-specific subgroups, thus limiting statistical power. This translates into another limitation concerning our lack of findings with respect to correlations with prodromal symptoms. It would have been promising to find a correlation between altered laterality and specific symptoms. Single shell data is another limitation. Multi-shell data would make the multi-compartmental fit more robust (86). Lastly, while our data are part of a longitudinal study with 1-year follow-up interviews, only four females converted to a first-episode of psychosis, which limits our ability to make predictions about conversion to psychosis.

Finally, one empirical next step to elucidate the association between a putative sex-specific neurodevelopmental disturbance in the frontoparietal network and verbal working memory dysfunction in the CHR state might be the inclusion of a sufficient number of individuals with a schizotypal tendency. In schizophrenia, as well as in individuals with schizotypy, atypical brain laterality is considered a potential underlying basis for the excess of non-right-handedness (i.e., mixed- and left-handedness) (87). Further, it has been shown that higher scores on schizotypy questionnaires have been previously able to predict conversion to psychosis in CHR subjects (88). Although our inclusion criteria involved schizotypal personality disorder, only one male was recruited. Therefore, we are not able to address this question in the present analysis.

In conclusion, the present study demonstrates that CHR females show greater rightward asymmetry of the SLF-III which we believe highlights the underappreciated role of sex in understanding white matter asymmetry and alterations in the pathophysiology of a high-risk state for developing psychosis. Thus, improving our understanding of both the sexual dimorphism of white matter asymmetry and the factors that influence these neurodevelopmental maturational processes, as well as the factors they influence, such as clinical and cognitive symptoms, may help to promote the development of novel interventions aiming to prevent transition into a chronic and difficult-to-treat disorder.

## Data Availability Statement

The raw data supporting the conclusions of this article will be made available by the authors, without undue reservation.

## Ethics Statement

The studies involving human participants were reviewed and approved by Ethik-Kommission - Ärztekammer Hamburg. The patients/participants provided their written informed consent to participate in this study.

## Author Contributions

CM and SS designed the study. JR, FN, and MM collected data. SS and ML analyzed data. TB, YR, and SC-K wrote codes. AL, GL, MK, FZ, LO'D, OP, and MS gave methodological support. SS, CM, and MS supervised the project. SS and AL worte the paper. All authors discussed the results and commented on the manuscript at all stages.

## Conflict of Interest

The authors declare that the research was conducted in the absence of any commercial or financial relationships that could be construed as a potential conflict of interest.
